# TGF-β1 Signaling: Immune Dynamics of Chronic Kidney Diseases

**DOI:** 10.3389/fmed.2021.628519

**Published:** 2021-02-25

**Authors:** Philip Chiu-Tsun Tang, Alex Siu-Wing Chan, Cai-Bin Zhang, Cristina Alexandra García Córdoba, Ying-Ying Zhang, Ka-Fai To, Kam-Tong Leung, Hui-Yao Lan, Patrick Ming-Kuen Tang

**Affiliations:** ^1^State Key Laboratory of Translational Oncology, Department of Anatomical and Cellular Pathology, The Chinese University of Hong Kong, Shatin, Hong Kong; ^2^Department of Applied Social Sciences, The Hong Kong Polytechnic University, Hung Hom, Hong Kong; ^3^Department of Nephrology, Tongji Hospital, Tongji University School of Medicine, Shanghai, China; ^4^Department of Paediatrics, The Chinese University of Hong Kong, Shatin, Hong Kong; ^5^Department of Medicine and Therapeutics, Li Ka Shing Institute of Health Sciences, The Chinese University of Hong Kong, Shatin, Hong Kong; ^6^Guangdong-Hong Kong Joint Laboratory on Immunological and Genetic Kidney Diseases, The Chinese University of Hong Kong, Shatin, Hong Kong

**Keywords:** transforming growth factor β, chronic kidney disease, renal inflammation, kidney fibrosis, immunity

## Abstract

Chronic kidney disease (CKD) is a major cause of morbidity and mortality worldwide, imposing a great burden on the healthcare system. Regrettably, effective CKD therapeutic strategies are yet available due to their elusive pathogenic mechanisms. CKD is featured by progressive inflammation and fibrosis associated with immune cell dysfunction, leading to the formation of an inflammatory microenvironment, which ultimately exacerbating renal fibrosis. Transforming growth factor β1 (TGF-β1) is an indispensable immunoregulator promoting CKD progression by controlling the activation, proliferation, and apoptosis of immunocytes via both canonical and non-canonical pathways. More importantly, recent studies have uncovered a new mechanism of TGF-β1 for *de novo* generation of myofibroblast via macrophage-myofibroblast transition (MMT). This review will update the versatile roles of TGF-β signaling in the dynamics of renal immunity, a better understanding may facilitate the discovery of novel therapeutic strategies against CKD.

## Introduction

Chronic kidney disease (CKD), an increasing contributor to morbidity and mortality, is predicted to become the 5th most common cause of death worldwide in 2040 (1, 2). CKD can be a primary disease or a complication initiated by other disorders, including glomerulonephritis (3), hypertension (4), diabetes (5), infection (6), and genetic causes (7). Its gradual development into end-stage renal disease (ESRD) is featured by the deposition of excessive extracellular matrix (ECM) and loss of kidney function (8). Unfortunately, current treatments are ineffective because of the complicated pathophysiological mechanisms of CKD. Despite there being multiple causes, it is well-accepted that CKD is a consequence of unresolved inflammation and renal fibrosis (9–14). Importantly, increasing evidence suggests the dysregulation of renal immunity is important for CKD development (15–17), e.g., promoting inflammation by their recruitment and adhesion to the renal epithelium (11, 18) and fibrosis by their secretome induced pro-fibrogenic responses respectively (17).

Transforming growth factor-beta (TGF-β) consists of 3 isoforms (TGF-β1, TGF-β2 TGF-β3), TGF-β1 is well-established as an indispensable driver of renal fibrosis in the pathogenesis of CKD, while the role of TGF-β2 and TGF-β3 remains largely undefined (11, 19–21). However, direct targeting of TGF-β1 signaling would affect its physiological functions in the regulation of cell differentiation, apoptosis, and immune homeostasis (22). Consequently, disease-specific pathogenic downstream of TGF-β1 pathway has been proposed to serve as an alternative therapeutic target and prognostic marker for CKD (23, 24). Recently, emerging studies have uncovered the downstream mechanisms of TGF-β1 in both adaptive and innate immunity during CKD. Better understanding of the regulatory mechanisms of TGF-β1 signaling in renal immunity may largely facilitate the therapeutic development of CKD (25).

## Importance of TGF-β1 in CKD Pathology

TGF-β1 plays an essential role in the pathogenesis of CKD due to its anti-inflammatory and fibrotic actions. TGF-β1 is well-demonstrated as an anti-inflammatory cytokine during the renal repair process at the early stage of kidney injury (26). In a mice model of crescentic glomerulonephritis, TGF-β1 inhibits the release of inflammatory cytokines as well as the infiltration of macrophages and CD3+ T cells for protecting injured kidney (27). TGF-β1 can promote the macrophages transiting from pro-inflammatory M1 into anti-inflammatory M2 phenotype (28). Nevertheless, short-term activation would facilitate the renal repair process, whereas endured activation would lead to renal fibrosis (15). Interestingly, TGF-β1 interrupts NF-κB pathway via Smad7 (29), interacts with β-catenin/Foxo complex (30), or modulates c-Jun N-terminal kinase signaling (31) to exert anti-inflammatory effect. In mice UUO and ischemic/reperfusion models, TGF-β1 also promotes β-catenin/T-cell factor (TCF) interaction, thereby simultaneously driving anti-inflammatory and pro-fibrotic responses via promoting β-catenin binding to Foxo and TCF, respectively (30, 31). Moreover, several studies further demonstrated the pro-fibrotic role of TGF-β1 signaling through mediating the ERK1/2 pathway, P38/MAPK pathway, and Akt/ERKs pathways (32, 33).

CKD would ultimately progress into end-stage renal disease (ESRD) due to the progressive fibrotic processes mediated by TGF-β1 signaling (34). TGF-β1 exerts its pro-fibrotic effects via both canonical (Smads dependent) and non-canonical (Smads independent) pathways. In the canonical pathway, Smad2 and Smad3 are two key downstream mediators of TGF-β receptor that are highly activated in renal fibrosis (35). Subsequently, activated Smad2 and Smad3 first complexed with Smad4 (36), then translocated into the nucleus to transcriptionally regulate pro-fibrotic molecules expression, including collagens, fibronectin, and alpha-smooth muscle actin (37–39), thereby facilitating fibrotic responses. However, each Smads protein is functionally distinct in the pathogenesis of CKD. Smad3 promotes while Smad2 suppresses CKD progression (40–42). Notably, Smad3 and Smad2 bind directly to the target gene, and Smad4 is lack of DNA-binding domains, but Smad4 still serve as regulators of the transcription process (43–47).

In the non-canonical pathways, TGF-β1 directly activates non-Smads signaling pathways, including MAPK pathway (48), PI3K/Akt/mTOR pathway (49), TGF-β1/p38 MAPK pathway (50), ILK (51), EGFR (52), and Wnt/β-catenin pathway (53). These non-canonical pathways largely contribute to the pathogenesis of renal fibrosis, including matrix formation (54), de-differentiation of proximal tubular cells (55), cell proliferation and migration (54), and apoptosis (56).

TGF-β1 signaling is the key mechanism of ECM synthesis by inducing myofibroblasts generation from number of origins, including epithelial cells, endothelial cells, resident fibroblasts, and pericytes. Epithelial to Mesenchymal Transition (EMT) is a well-characterized pathological process of renal fibrosis featured by the conversion of epithelial cells into mesenchymal phenotypes. TGF-β1 signaling drives key events of EMT *in vivo* and *in vitro*, including loss of epithelial adhesion, *de novo* α-SMA expression, and cell migration (57, 58). During EMT, the migratory ability and mesenchymal markers, fibronectin, and α-smooth muscle actin (α-SMA) were acquired, while epithelium adhesion and E-cadherin protein were lost after the transition (59–61). Thus, EMT contributes to the pathogenesis of kidney fibrosis via direct generation of the collagens producing myofibroblasts (62). In the canonical pathway, Smad3 is highly activated in the UUO kidney *in vivo*, and TGF-β1 treated renal tubular epithelial cells *in vitro*, driving EMT for the myofibroblast generation and associated kidney fibrosis, which is blocked by Smad3 deletion and TGF-β1 neutralizing antibody (63–65). Non-canonical pathways, including MAPK, Rho-like GTPase, PI3K/Akt, and Wnt signaling, have been illustrated to have played emerging roles in EMT induction (28, 66, 67). TGF-β1/Smad3 signaling also drives Endothelial to Mesenchymal transitions (EndoMT), where smad3 inhibitor and endothelium-specific TGF-β receptor knockout reduces EndoMT mediated diabetic nephropathy in streptozotocin (STZ)-induced diabetes and tubulointerstitial fibrosis in unilateral ureteral obstruction models *in vivo* (68, 69). Resident fibroblasts and pericytes are rich sources of myofibroblasts, demonstrated by lineage tracing studies with P0-Cre and Foxd1-Cre to label myofibroblasts derived from fibroblasts and pericytes, respectively (70, 71). Resident fibroblasts and pericytes were activated into α-SMA^+^ myofibroblasts in mice model of obstructive kidney fibrosis via TGF-β1/Smad3 signaling (72–74). Therefore, TGF-β1 activates various cell types via both of the canonical and non-canonical pathways, generating myofibroblast for excess ECM deposition, ultimately contributing to fibrotic responses in CKD.

## TGF-β1 in Adaptive Immunity of CKD

### B Cell

Interestingly, dysregulation of humoral immunity was observed in ESRD patients; only 65% of ESRD patients can produce sufficient titer of antibodies upon vaccination, in contrast to the 95% in healthy control (16, 75). A previous study demonstrated that B1 (CD19+CD5+) and B2 lymphocytes (CD19+CD5–) are negatively associated with the progression of CKD but positively correlated with the survival of elderly CKD patients, suggesting B cell deficiency could be a prognostic factor of CKD progression (76). Autoantibodies production by B-cells is crucial for the development of IgA nephropathy and lupus nephritis. In the pathogenesis of IgA nephropathy, B-cells produce aberrant galactosylated IgA and its autoantibodies (anti-glycan antibodies) to form immune complexes, which deposition on mesangial cells to initiates glomerulonephritis and subsequent CKD progression (77–79). Similarly, in Lupus nephritis, multiple autoantibodies were involved in the immune complexes formation, including anti-dsDNA (80), anti-C1q (81), and anti-nucleosome (82) autoantibodies. Mechanistically, TGF-β suppresses B-cell maturation into antibody-producing cells, resulting in antibody abnormalities or autoantibodies production (83, 84). TGF-β1 inhibits pre-B cell proliferation via suppressing PI3K/Akt signaling and induces a cell cycle arrest of pre-B cells specifically at the G0/G1 phase (85). TGF-β1 also hinders B cell proliferation and activation indirectly via contacting the regulatory T cells, associated with the upregulation of granzyme A, granzyme B, and perforin (86). TGF-β1 induces B cell-activating factor (BAFF) production from the macrophages via Smad3/4 and PKA/CREB signaling pathways (87). BAFF is a key cytokine regulating B-cells activity, including proliferation, differentiation, apoptosis, and immunoglobulin secretion; excessive BAFF would suppress B-cell development resulting in autoantibodies production in IgA nephropathy and Lupus nephritis (83, 84, 88) Taken together, TGF-β1 suppress B lymphocytes development in the pathogenesis of kidney diseases via both direct and indirect mechanisms.

### T Cell

T lymphocyte infiltration has been observed in CKD biopsies (89, 90) and is positively correlated with the deterioration in glomerular filtration rate (91), indicating a pathogenic role of T lymphocytes in the pathogenesis of CKD. Interestingly, CD8+ T cell abundance is significantly associated with the TGF-β1 level in the kidney biopsies of lupus nephritis (92). In a mice model of Crescentic Glomerulonephritis (GN), CD3^+^ T cell infiltration and associated glomerular and tubulointerstitial injuries were largely suppressed in latent TGF-β1 transgenic mice, compared with wildtype mice (93). TGF-β1 plays a crucial role in the modulation of T cell migration, activation, proliferation, and death. The recruitment and differentiation of CD4^+^ T cells were regulated by mesenchymal stem cells (MSCs) via TGF-β1 signaling (94) while TGF-β1 enhances CD8^+^ T-cell activation and proliferation by switching the immune-suppressive myeloid-derived suppressor cells (MDSCs) into immune-stimulating phenotype in a SMAD-2 dependent manner (95). This may explain CD8^+^ T-cell tubulitis and associated TGF-β1/Smad2/3 signaling activation in a rat model of aristolochic acid nephropathy (AAN) (96). In addition, TGF-β1 induces oxidative stress in injured renal tissue via mitochondrial and NADPH oxidases ROS production and suppression of antioxidant system (97–99). In Mercuric chloride intoxication and Dahl salt-sensitive rat models, elevated ROS level leading to the interstitial CD8+ T cells infiltration and associated tubular damage (100, 101). Adoptive transfer of oxidizing agents treated CD4+ T cells also caused immune complex glomerulonephritis in syngeneic recipient mice (102).

On the other hand, regulatory T cells (Tregs) play a protective role in CKD by suppressing inflammation and immune cell-mediated fibrosis (30, 103–106). Notably, abundance of peripheral Tregs is significantly reduced in CKD patients compared to the healthy controls (107). TGF-β1 is well-characterized as a Tregs inducer (108, 109). TGF-β1 has been demonstrated to increase the proliferation, differentiation, and function of Tregs by not only up-regulating Foxp3 (a master transcription regulator of Tregs) expression via PP2A pathway (110) but also suppressing IL-12R (111). Furthermore, TGF-β1 induces membrane-bound TGF-β1 on the Treg cells to suppress naive CD4+ T cells expansion for immune suppression via activating Smad3 (112). Surprisingly, Tregs are able to convert into TGF-β1-producing cells in the inflammatory environment, which markedly up-regulates the level of TGF-β1 in UUO-obstructed kidney, therefore aggravating chronic inflammation and renal fibrosis (113).

### TGF-β1 in Innate Immunity of CKD

#### Neutrophil

Neutrophils are well-documented because of their aggravating role in inflammation (114), where neutrophil-to-lymphocyte ratio is a popular prognostic marker for estimating the mortality of CKD patients (115). Neutrophils can initiate and amplify inflammatory responses by releasing pro-inflammatory cytokines (114, 116), and serves as a rich source of TGF-β1 in inflamed tissues (117, 118). During inflammation, TGF-β1 facilitates the accumulation of neutrophils (119, 120), therefore inhibiting TGF-β1 effectively alleviates neutrophil infiltration and inflammation (121). Furthermore, TGF-β1 signaling can be blocked by preventing Smad3 activation, which has been proposed as a potential therapeutic strategy for fibrotic diseases driven by neutrophil-mediated inflammation (122, 123).

### Dendritic Cell

Dendritic cells (DCs) facilitate renal inflammation via promoting CD8+ T cell proliferation and activation during the development of CKD (124, 125). Mechanistically, TGF-β1 promotes DCs accumulation in fibrotic tissue (126) and modulates DCs-mediated proliferation and activation of T cells (127–130), contributing to the imbalance between Th17 and Treg (131) and the interleukin 17 (IL-17) release from naive CD4^+^ cells (132). Importantly, TGF-β1 further stimulates TGF-β1 release from DCs in an autocrine manner, serving as a major source of TGF-β1 in the tissue biopsies from stage IV–V CKD patients (133, 134) and suppressing inflammatory cytokines (IL-12, IL-18) production in DCs (135, 136). These findings suggest DCs can regulate the proliferation, activation, differentiation, and function of T cells via TGF-β1 signaling during inflammation. It has been demonstrated that targeting of DCs maybe able to suppress CKD progression by attenuating renal inflammation and fibrosis (94, 137, 138).

### Macrophage

Macrophage is a key player in the pathological process of CKD that their infiltration due to their pathogenic actions in both renal inflammation and fibrosis (15, 16, 87, 89, 139, 140). It has been reported that TGF-β1 participated in macrophages-mediated immune dysfunction during the progression of CKD (15, 141, 142). TGF-β1 largely increases macrophages infiltration and accumulation in the injured kidney via stimulating the release of a potent cytokine for macrophages recruitment monocyte chemoattractant protein-1 (MCP-1) from various types of renal cells (143–145). TGF-β1 also regulates macrophage polarization and immunomodulatory cytokines secretion. Upon the kidney injury, TGF-β1 transits M1 macrophage into regulatory M2c phenotype to facilitate kidney repair by producing the immunosuppressive and matrix remodeling activities (146–148). However, the CCL18 secreted from these CD163^+^ macrophages also promotes fibroblast proliferation, leading to the acceleration of kidney fibrosis (149). TGF-β1 also induces the expression of B cell-activating factor (BAFF), a key regulator of B cell activities, in macrophages via Smad3/4 dependent mechanism to influence the macrophages-mediated pathogenic function of B cells (87). The elevated plasma level of BAFF was observed in ESRD patients compared to the control group (150–152). Interestingly, the interaction between macrophages and TGF-β1 is mutual, where macrophage is the effector and a rich source of TGF-β1, actively producing and secreting TGF-β1 in inflamed kidney tissue (153, 154). Thus, blockade of TGF-β1 signaling effectively reduces macrophages infiltration (41, 155, 156) as well as significantly reduces macrophage polarization and extracellular matrix deposition (157, 158).

### Novel Fibrotic Mechanism of TGF-β1: Macrophage-Myofibroblast Transition

**Figure 1:**
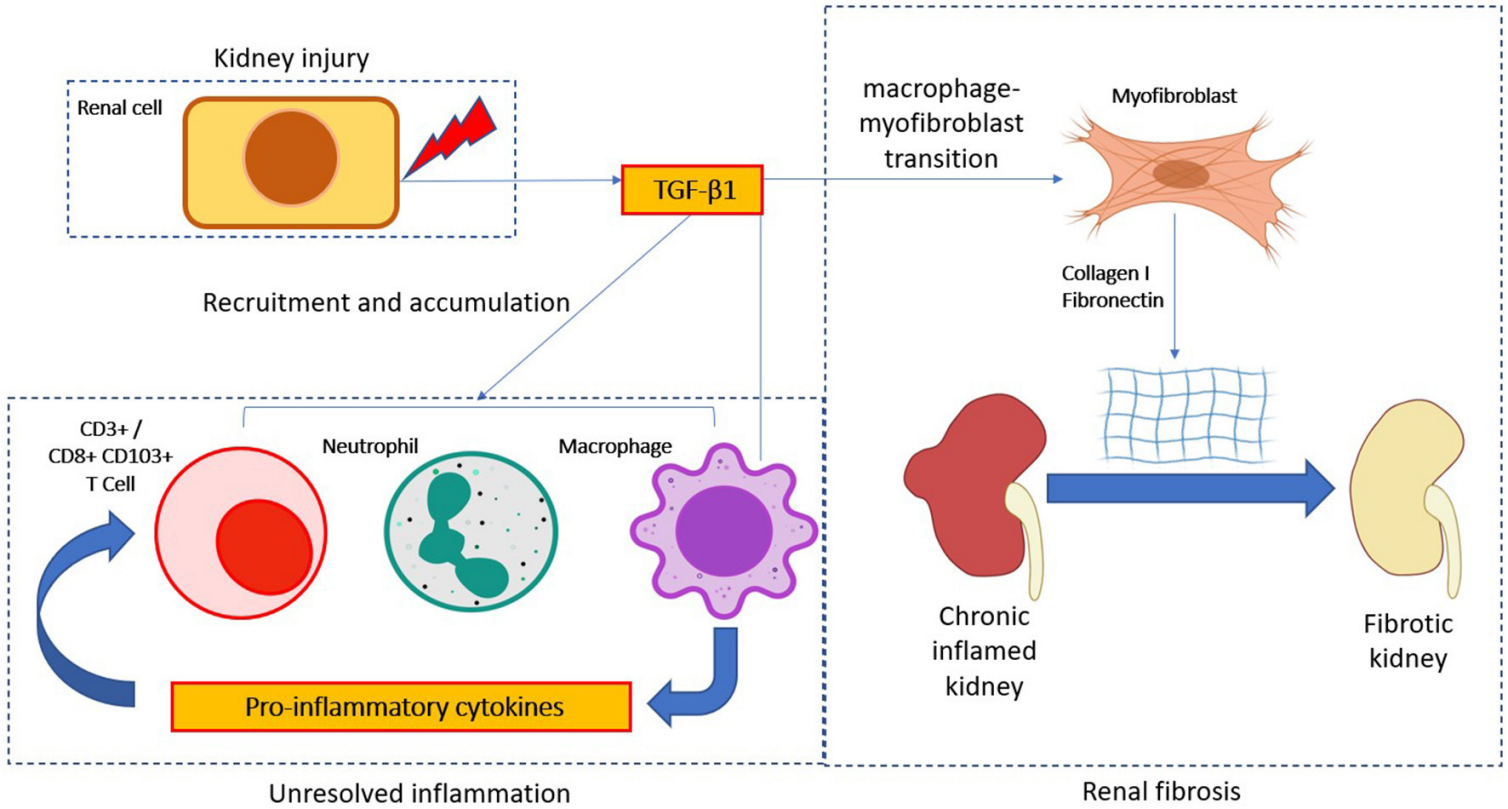
TGF-β1 in immune cell mediate CKD progression. TGF-β1 modulates immune cell activity in the progression of chronic kidney disease (CKD). After kidney injury, TGF-β1 is released by kidney cells to aid the resolution of inflammation. However, persistent TGF-β1 signaling activation would promote a chronic inflammation state via amplification of inflammatory responses. Notably, chronic TGF-β1 signaling activation would further transdifferentiate macrophage into myofibroblast to produce excessive extracellular matrix molecules (Collagen I and fibronectin), thus eventually lead to the pathogenesis of CKD.

Myofibroblast is an important effector cell type that contributes to the switching of unresolved inflammation to be renal fibrosis, they featured by a high level of α-SMA expression and excessive extracellular matrix deposition (159). The sources of pathogenic myofibroblasts are highly heterogeneous and still largely unclear and controversial (160, 161). Macrophage-myofibroblast transition (MMT) is a newly-identified phenomenon driven by TGF-β1 signaling as a direct mechanism of macrophage for promoting myofibroblast generation under unresolved renal inflammation (15, 162, 163) (Figure 1). Mechanistically, TGF-β1/Smad3 signaling is suggested as the key regulator for initiating MMT during renal fibrosis in a UUO model *in vivo*, where TGF-β1 induces the *de novo* expression of myofibroblast marker α-SMA and effector collagen I in the bone marrow derived macrophages (BMDMs) via a Smad3-dependent mechanism (164). Bioinformatic analysis of TGF-β1/Smad3 dependent transcriptome of MMT *in vitro* further reveals Src and Pou4f1 as the pathogenic mediator in the Smad3 downstream signaling, representing a precise therapeutic target for blocking MMT (24, 165). In brief, TGF-β1/Smad3 directly activates a Src-centric gene network in BMDMs via transcriptional regulation for promoting the MMT process in the fibrosing kidney (15). More importantly, Tang et al. further discovered the importance of a neural-specific homeobox/POU domain protein Pou4f1 in the Smad3 downstream as a specific mediator for regulating MMT (24). Besides, non-canonical TGF-β1 signaling also induces MMT *via* β-catenin/TCF pathway, promoting pro-fibrotic gene expression in the kidney infiltrating macrophages (30, 166). Inhibitor of Src (PP1) and TCF (ICG-001) and BMDM-specific Pou4f1 silencing effectively suppress the MMT process and associated renal fibrosis, suggesting MMT may be therapeutically targeted to restrain CKD progression (24, 165).

## Therapeutic Strategies for Targeting the TGF-β1-Mediated CKD

**Figure 2:**
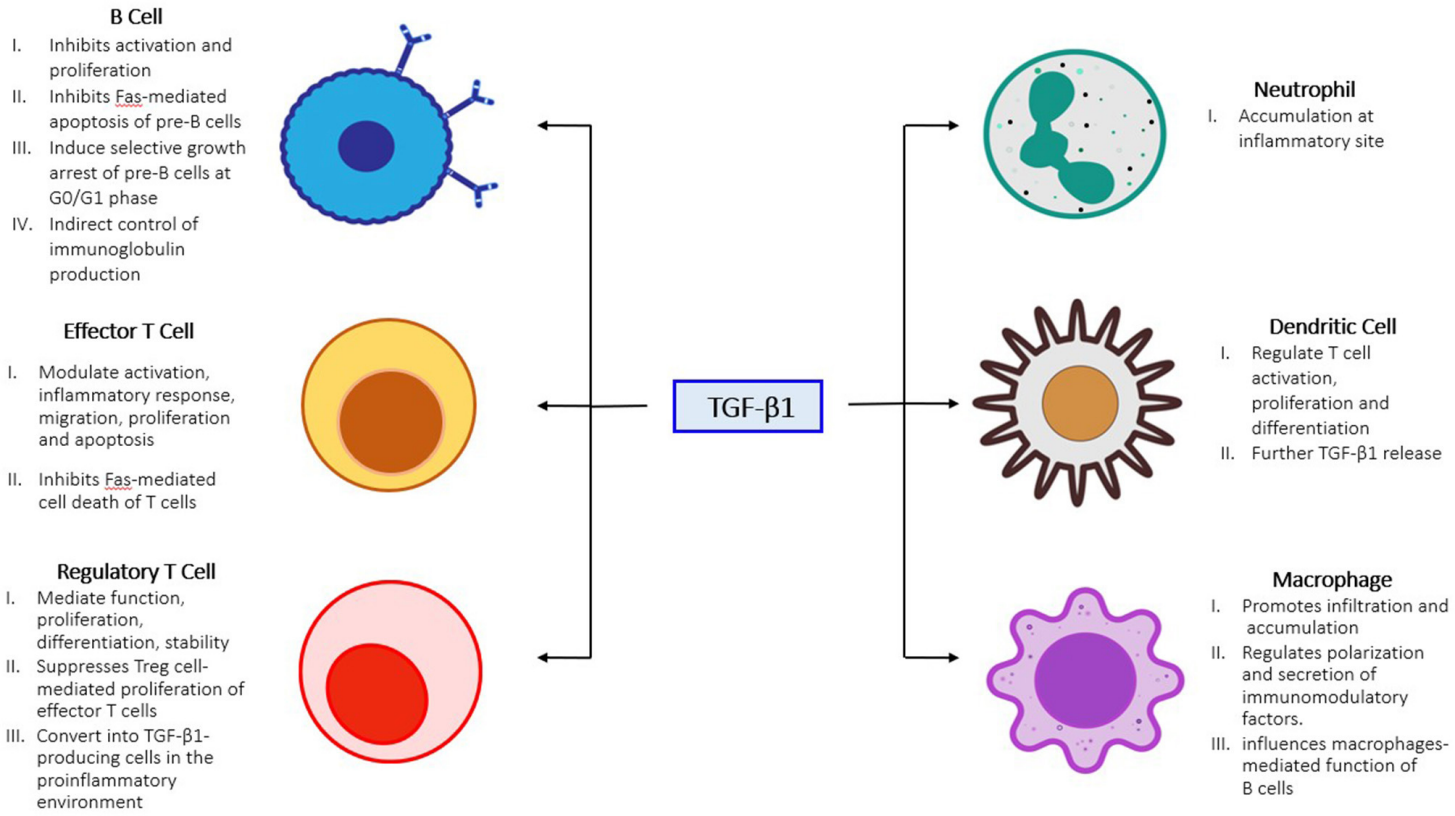
Role of TGF-β1 in unresolved inflammation. TGF-β1 activation promotes innate immune cells accumulation (neutrophil, macrophage, and dendritic cell) at the inflammatory site, which in-turn further activate adaptive immune cells (B and T cells) and dendritic cells to amplify the inflammatory responses in chronic inflamed kidney.

TGF-β1 signaling is essential for the progression of renal fibrosis and has been proposed as a therapeutic target for CKD (Figure 2), however systematically targeting TGF-β1 would also suppress its physiological functions and may result in adverse side effects (167, 168). Emerging clinical trials demonstrated that direct targeting TGF-β1 signaling was highly associated with adverse events in 23 to 87% of the kidney patients (167, 169, 170). Nevertheless, alternative approaches that specifically targeting the pathogenic mediators in TGF-β1 downstream may prevent the side effects. The molecular mechanism of Smad3 in renal pathology is intensively elucidated among the other Smads, genetic deletion of Smad3 effectively protected mice against collagen deposition after kidney injury (63, 171, 172). Therefore, several strategies targeting Smad3 have been investigated in a number of pre-clinical studies. Encouragingly, a Smad3 specific inhibitor SIS3 and a natural compound isolated from Poria *cocos* Poricoic acid effectively suppressed renal fibrosis development in experimental models of diabetic nephropathy (68), obstructive nephropathy (173), and ischemia-reperfusion injury (174) *in vivo*. In addition, diterpene and triterpenes (175), 25-O-methylalisol F (176), and IC-2 derivatives (177) are also capable of suppressing Smad3 activation and pro-fibrotic molecules production (Collagen I and fibronectin) in the renal epithelial cells. Importantly, emerging evidence showing macrophages mediate the therapeutic effect of Smad3 inhibition. Smad3 inhibition or genetic deletion suppressed MMT in mouse models of chronic Renal Allograft Injury (178), unilateral ureteric obstruction (164), contributed 50–60% reduction of myofibroblast population, and suppressed macrophage infiltration in type 2 diabetic nephropathy (179), thus contributing to the protective effect of Smad3 targeted therapy. Furthermore, noncoding RNAs including LRNA9884 (180), Erbb4-IR (20, 181), miR-29b (182), anti-miR-433 (183), lnc-TSI (184), and anti-miR-21 (185) were discovered from the TGF-β/Smads signaling for the obstructive and diabetic nephropathy. Among them, RNA therapies targeting LRNA9884 and miR-29b could modulate leukocytes infiltration via inflammatory cytokines expression, thus suppressing renal inflammation in diabetic nephropathy (180, 182, 186, 187). Importantly, these RNA-based therapies effectively restrained CKD progression with minimal side effects thanks to their specificity (188, 189). In addition, targeting the non-canonical TGF-β1 signaling including ERK1/2 (190, 191), β-catenin (192), p38 (193), and PI3K/Akt (49) also suppressed the pro-fibrotic actions in obstructive nephropathy, demonstrating the therapeutic potential of targeting the TGF-β1 downstream mediators (Table 1).

**Table 1:** Pre-clinical studies for the treatment of CKD by specifically targeting the downstream of TGF-β1.

**Drugs**	**Target**	**Route and effective dose**	**Disease model**	**Results**	**References**
**Canonical pathway**					
SIS3	Smad3	I.p. 0.2, 2 mg/kg/day	UUO kidneys 1 week BALB/c male mice	↓ Fibrosis ↓ p-Smad3/Fn/Collagen I/III ↓ Myofibroblast (α-SMA^+^ cells)	(173)
SIS3	Smad3	*In vitro* 1 μM I.p. 2.5, 5 μg/g SIS3	TGF-β1/AGEs induced Mouse pancreatic microvascular endothelial cells (MMECs) 5 Days STZ 50 μg/g induced diabetes on Tie2-Cre; Loxp-EGFP mice (C57BL/6J)	↓ p-Smad3 ↓ RAGE-mediated EndoMT ↓ Collagen I/ α-SMA/ Fn	(68)
Poricoic Acid A (PAA)	Smad3	*In vitro* 10 μM 10 mg/kg oral gavage	TGF-β/ hypoxia/reoxygenation treated HK-2 cells Rats IRI model	↓ p-Smad3 ↓ Collagen I/ α-SMA/ Fn	(174)
IC-2 derivatives	Smad3	*In vitro* 10, 20 μM	TGF-β1 induced Tubular epithelial cells HK-2 cells	↓ p-Smad3 ↓ Collagen 1	(177)
25-O-methylalisol F (MAF)	Smad3	*In vitro* 10 μM	TGF-β1/ANG stimulated NRK-52E cells Tubular epithelial cells	↓ p-Smad3 ↓ Wnt/β-catenin ↑ Smad7 expression ↓ Collagen I, Fn, α-SMA	(176)
Diterpene (PZF) and triterpenes (PZH)	Smad3	*In vitro* 10 μM	TGF-β1/ANGII induced Human kidney proximal epithelial cells (HK-2) Immortalized mouse podocytes (MPC5)	↓ p-Smad3 ↓ Collagen I/ α-SMA/ Fn ↓ Wnt/ β-catenin ↓ MMP-7/PAI-1/Fsp-1	(175)
miR-29b	Smad3	Ultrasound microbubble mediated-Mir-29b gene transfer	db/db or db/m mice AGE induced rat MC line and tubular epithelial cell line (NRK52E)	↓ p-Smad3/ Collagen I/III ↓ Microalbuminuria ↓ Mesangial index (histological injury)	(182)
Anti-miR-433	Smad3	Ultrasound-mediated gene transfer of inducible miR-433 shRNA	Obstructive nephropathy mouse model (UUO) Normal rat TEC line, NRK52E	↓ Collagen I/ α-SMA/ Fn ↓ p-Smad3	(183)
lnc-TSI	Smad3	i.v. injection of pcDNA3.1-lnc-TSI	UUO rat model TGF-β1 treated human TECs	↓ Collagen I/ α-SMA/ Fn ↓ Kidney fibrosis (tubular interstitial fibrosis indexes/Serum creatinine)	(184)
Anti-miR-21	Smad3	Ultrasound-mediated gene transfer of inducible miR-21 knockdown	High glucose-induced rat mesangial cell (MC) and tubular epithelial cell (TEC), NRK52E Kidneys of db/db mice	↓ Collagen I/ IV/ Fn ↓ p-Smad3	(185)
**Non-canonical pathway**					
Trametinib (MEK inhibitor)	ERK1/2, mTORC1	3 mg/kg oral gavage	UUO mouse model	↓α-SMA/ Vimentin ↓ p-ERK1/2, p-Akt	(191)
Renalase	ERK1/2	Adenovirus renalase gene delivery	UUO mouse model	↓ p-ERK1/2 ↓ Collagen I/ α-SMA/ Fn	(190)
QiShenYiQi (QSYQ) Traditional Chinese Medicines	β-catenin	250, 500 mg/kg/d intra-gastric *In vitro* 5, 10, 20 μg/ml	UUO rat model TGF-β treated Normal kidney proximal tubular (NRK52E) and renal fibroblast cells (NRK49F)	↓ Collagen I/ α-SMA/ Fn ↓ β-catenin	(192)
α1-adrenoceptor inhibitors	p38	Tamsulosin (i.p.) 0.4 mg/kg/day	UUO mouse model	↓ Serum creatinine and urea ↓ KIM-1/NGAL/ PAL-1 ↓α-SMA/vimentin/Snai1/ Fibronectin	(193)
Aloe-emodin	PI3K/Akt/mTOR	20 mg/kg/day oral gavage	UUO mouse model	↓ Tubule injury index score. ↓ Masson trichromatic +ve area ↓ Collagen I/Fn ↓ Scr/BUN/urine volume	(49)

*UUO, unilateral ureteral obstruction; EMT, epithelial-mesenchymal transition; SIS3, specific Inhibitor of Smad3; CKD, chronic kidney disease; Fn, Fibronectin; Scr, Serum creatinine; BUN, blood urea nitrogen; α-SMA, Alpha-smooth muscle actin; STZ, Streptozotocin; ANG, Angiotensin; KIM-1, Kidney Injury Molecule-1; NGAL-1, neutrophil gelatinase-associated Lipocalin; PAL-1, plasminogen activator inhibitor 1, RAGE MMP-7/PAI-1/Fsp-1*.

## Conclusion and Future Perspectives

TGF-β1 exerts its pathogenic roles in the progression of CKD by regulating both of the innate and adaptive immunity in the injured kidney via the canonical and non-canonical pathways including a novel fibrotic mechanism MMT. The TGF-β1 driven development of renal fibrosis from unresolved inflammation is well-observed, but underlying mechanisms remain largely unexplored. Better understanding of the underlying mechanisms of TGF-β1 pathways uncovered a number of novel pathogenic mediators from the downstream signaling, which may represent an effective therapeutic strategy to prevent renal inflammation progress into fibrosis. Moreover, the TGF-β1 regulating immune cells also contribute to other fibrotic diseases. In addition, further studies of TGF-β isoforms (TGF-β2, TGF-β3) on immune cells may reveal their therapeutic potential in renal immunity driven CKD progression. Current clinical trials targeting renal immunity shows promise, further investigation for validating the safety and effectiveness of these therapeutic approaches would discover new hope for patients with fibrotic diseases in the coming future.

## Author Contributions

PT, AC, C-BZ, CG, and Y-YZ responsible for literature research and writing. K-FT, K-TL, and H-YL reviewed the manuscript and made significant revisions on the drafts. PT supervised and finalized of this work. All authors have read and agreed to the published version of the manuscript.

## Conflict of Interest

The authors declare that the research was conducted in the absence of any commercial or financial relationships that could be construed as a potential conflict of interest.
